# Preparation of Glass Fabric/Poly(l-lactide) Composites by Thermoplastic Resin Transfer Molding

**DOI:** 10.3390/polym11020339

**Published:** 2019-02-15

**Authors:** Elodie Louisy, Fabienne Samyn, Serge Bourbigot, Gaëlle Fontaine, Fanny Bonnet

**Affiliations:** 1 Univ. Lille, CNRS, INRA, ENSCL, UMR 8207, UMET, Unité Matériaux et Transformations, F-59000 Lille, France; elodie.louisy@univ-lille.fr (E.L.); fabienne.samyn@ensc-lille.fr (F.S.); serge.bourbigot@ensc-lille.fr (S.B.); gaelle.fontaine@ensc-lille.fr (G.F.); 2 Univ. Lille, CNRS, Ecole Centrale, Univ. Artois. ENSCL, UMR 8181, UCCS, Unité Catalyse et Chimie du Solide, F-59000 Lille, France

**Keywords:** composites, resin transfer molding, polylactide, ring opening polymerization, tin octoate

## Abstract

This study reports the first example of the production of polylactide composites prepared by Thermoplastic Resin Transfer Molding (T-RTM) via in situ bulk polymerization of l-lactide (l-LA) after injection in a closed mold containing glass fabrics. Tin octoate Sn(Oct)_2_ was used as the catalyst and first evaluated at the lab-scale in the experimental conditions required in the tank and in the mold of the RTM device. The reactions were then upscaled in the RTM in the absence of reinforcement to ensure the feasibility of the process (transfer and polymerization). Finally, poly-l-lactide (PLLA)-based composites with glass fabrics as the reinforcement were obtained. The resulting PLLA matrices exhibited conversions up to 99% along with high molar masses of up to 78,000 g·mol^−1^ when the polymerization was carried out under dynamic vacuum (vacuum-assisted RTM, VARTM). Moreover, a good impregnation of the glass fabrics by the matrix was observed by optical microscopy.

## 1. Introduction

Composite materials display many advantages over traditional materials, both in terms of lightness and mechanical or chemical resistance [1]. The development of composites meets the requirements of industrial markets for applications in the fields of transport, construction, sports, and leisure. In the context of sustainable development, a growing number of studies are focusing on the development of bio-based composites [2]. The innovations regarding these new materials are based on the use of vegetable fibers as the reinforcements and the development of bio-based resins as the polymer matrices. Among the various bio-sourced materials, polylactide (PLA) is a biodegradable and biocompatible polymer [3] that has become a major actor on the market, in particular for biomedical and short-lifetime applications [4]. PLA displays some intrinsic characteristics, such as low *T*_g_ (around 60 °C) and low elongation at break (2–6%), which limit its use in various applications. In order to overcome these problems and to allow PLA to be used for a wider range of sustainable long-lifetime applications, it is commonly used in the form of blends with other polymers [5] or in composites [6]. Regarding composites, there are three main types of processes for their manufacture: open molding, closed molding, and cast polymer molding. Within these molding categories there exists a variety of processing methods, each of them displaying their own benefits [7]. Among closed molding processes, Resin Transfer Molding (RTM) is an innovative process based on the injection, in a mold containing a reinforcement, of a monomer and a catalyst in order to carry out the polymerization of the matrix in situ [8,9]. The major advantages of this method over conventional melt processes is the possibility of using a high fiber content while improving their wetting by the matrix and producing parts displaying two smooth surfaces. The RTM process is commonly used for the high-volume production of net shape structural parts using low-viscosity thermoset resins. The most widely used resins are polyurethane, ester and epoxy resins [10]. Although a wide range of thermosetting matrix resins are available on the market, there are few commercial resins for thermoplastic matrices, namely polyacrylate matrices (Arkema Elium), polyamides (AP-Nylon Caprolactam from BASF) or polybutylene terephthalates (Cyclics CBT from Ems-Chemie AG). Regarding academic work on bio-based thermoplastic composites manufactured by RTM process, prototypes with poly(ε-caprolactone) as the matrix were reported [11,12], the ε-caprolactone monomer being liquid at room temperature and thus easy to handle. Concerning lactide monomer, no example of PLA composite produced by RTM process has been reported to date. Lactide is a solid, and thus needs to be heated to melt prior being injected in the mold, which could be an issue as it is already mixed with the catalyst in the injection tank and so the polymerization can be initiated. This requires the selection of suitable polymerization catalysts to meet the specific constraints of the RTM process. Nevertheless, this work presents the possibility of producing glass fabric/poly(l-lactide) (PLLA) composites by Thermoplastic Resin Transfer Molding (T-RTM) with the use of tin octoate, Sn(Oct)_2_, as the catalyst. The three-step approach followed to define processing conditions (*i.e*., temperature, catalyst loadings, reaction time) suitable for the preparation of PLLA-based composites is presented. The experimental conditions were first optimized on the basis of small lab-scale experiments before the scale-up in the T-RTM in the absence of reinforcement to validate them, and finally the glass fabric/PLLA composites were prepared. 

## 2. Materials and Methods

### 2.1. Reagents and Apparatus

**Figure 1:**
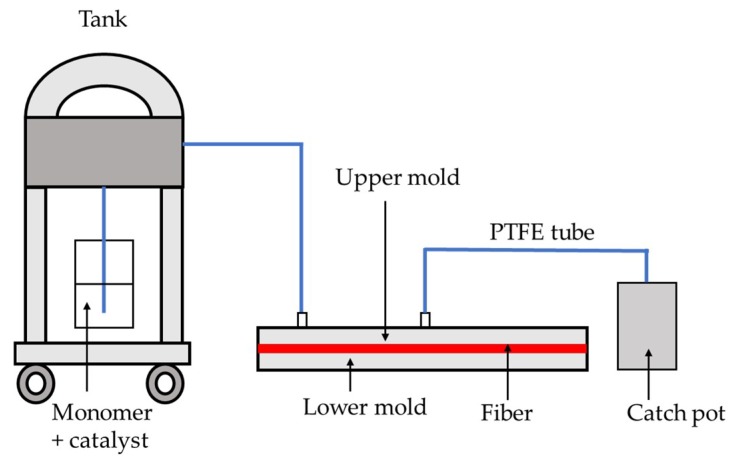
Resin transfer molding (RMT) device.

l-lactide (l-LA) was supplied by Purac (Gorinchem, The Netherlands), preserved in sealed bags and used as received (H_2_O content, measured by Karl-Fischer analysis, (Bethune, France), was found inferior to 5 ppm). The catalyst, tin octoate (Sn(Oct)_2_; purity between 92.5% and 100.0%) was supplied by Sigma-Aldrich (Darmstadt, Germany). The glass fabrics, HexForce ® E GLASS FABRIC 01113 1250 TF970, supplied by Hexcel (Stamford, CT, USA), were dried in an oven at 70 °C for 24 h prior use. The RTM apparatus (CIJECT III injection tank with a capacity of 7.5 L for 7 bars) (Figure 1) and the molds were supplied by DIATEX (Lyon, France). Two types of molds were used: a one-imprint 120 mm × 120 mm × 5 mm conduction-heated mold used under press (mold 1) and a two-imprint 120 mm × 120 mm × 5 mm self-heating mold (mold 2) (Figure S1 in the SI).

### 2.2. Bulk Polymerization at the Lab Scale

Small-scale bulk polymerization tests were performed in a 20 mL glass reactor. The l-lactide (1 g) and the catalyst were weighed in a glove box with monomer-to-catalyst ratios [l-LA]/[Sn] from 500 to 5000. The reagents were mixed in the reactor, which was then sealed with a plug and grease before being taken out of the glove box. The reactor was then immersed in an oil bath at the desired reaction temperature. The stirring was done using a magnetic stirrer. At the end of the reaction, the highly viscous medium was dissolved in chloroform. A small amount of the solution was sampled and dried under vacuum to determine the conversion by ^1^H NMR analysis in CDCl_3_ (see Figure S2 in the SI). Finally, the solution was poured dropwise in ethanol, and the polymer was filtered and dried under vacuum before performing SEC analysis.

### 2.3. Preparation of PLLA Matrices and Glass Fabric/PLLA Composites by T-RTM

The sealed bags containing the l-lactide (mass = 100 g for mold 1 and 400 g for mold 2) were open just before use. The monomer and the catalyst were mixed in an aluminum pot, which was then placed into the RTM injection tank which was heated to 120 °C beforehand. The mixture was heated under vacuum in the tank until it was completely melted (30 or 45 min for 100 or 400 g of l-LA, respectively) and was then mixed for 1 min under stirring. The injection of the mixture into the mold, through PTFE tubes (4 mm × 6 mm) supplied by DIATEX, was carried out by a flow of nitrogen under a pressure of 1 to 2 bars, and was complete in all cases after 2 min. When the experiment was conducted by vacuum-assisted resin transfer molding (VARTM), during the injection step under nitrogen, a vacuum of -1 bar was applied at the mold outlet, through a PTFE tube, using a ECVP425 vacuum pump supplied by Easy composite (Staffordshire, UK). When the mixture flowed into the PTFE outlet tube, the latter was blocked with a metal clamp. The vacuum of the pump was interrupted once the outlet mixture solidified in the PTFE tube due to the cooling. 

### 2.4. Liquid ^1^H NMR

^1^H NMR spectra of the polylactides synthesized at the lab scale or by RTM process were recorded on a Bruker Avance 300 MHz instrument at 300 K in CDCl_3_ supplied by Sigma-Aldrich (purity of 99.8%). The samples (about 5–15 mg) were dissolved in 0.5 mL of CDCl_3_ in a tube with 5 mm diameter. The scan number was set to 32 and the delay (D1) between each scan was set at 4 s. The conversion of the reaction was determined by integration of the CH signals of both the residual monomer and the polymer at 5.05 and 5.15 ppm, respectively (see Figure S2 in the SI).

### 2.5. SEC Measurement

The number-average molar masses (*M*_n_) and dispersities (*Ð* = *M*_w_/*M*_n_) of the polylactides obtained at the laboratory scale were determined by size exclusion chromatography (SEC) in THF (99+%, extra pure (stabilized with BHT) supplied by Acros Organics (Geel, Belgium) at 40 °C (1 mL/min)) using a triple detection system equipped with an Alliance Waters e2695, a multiangle light scattering detector (MALS, Wyatt Technology mini DAWN TREOS, Santa Barbara, CA, USA), and a refractive index detector (Waters 2414). The SEC system was equipped with three Waters Styragel (HT1, HT3, and HT4) columns. The differential refractive index (DRI) increment (dn/dc) value for the linear polylactide is 0.0558 mL·g^−1^ in THF at 40 °C (polymer handbook). Samples were prepared by dissolving the product (~10 mg) in 4 mL of THF. The solutions were then filtered with 0.45-μm filters. 

The number-average molar masses and dispersities of the polylactide matrices synthesized by the RTM process were determined by size exclusion chromatography in CHCl_3_ at 20 °C with a triple detection system, equipped with a multiangle light scattering detector (Wyatt TREOS) and a refractive index detector (Schimadzu RID 10A, Marne-la-Vallée, France). The SEC system was equipped with three PL gel MIXED C (300 mm × 7.5 mm polystyrene/divinylbenzene) columns from Aligent, (Santa Clara, CA, USA). The MALS method was used to determine the absolute molar masses of the PLA. The dn/dc used for PLA in chloroform was 0.023. Samples were prepared by dissolving the product (2–5 mg) in 1 mL of CHCl_3_. The solutions were put under stirring for one night, and then filtered with 0.45-μm filters. See the chromatogram of run 15 in Figure S3 in the SI.

### 2.6. DSC Measurements

The glass transition temperature (*T*_g_) and the melting temperature (*T*_m_) of the PLLA matrices were determined by differential scanning calorimetry (DSC) with a TA Discovery instrument (New Castle, DE, USA). During the analysis, a small amount of polymer (5–15 mg) was introduced into an aluminum pan that was closed with a lid. The analysis was carried out under nitrogen flow (50 mL/min). The samples were heated from 25 to 200 °C with a heating rate of 10 °C/min, then cooled from 200 to 25 °C at the same rate. Two heating and cooling cycles were carried out. Thermal characteristics were determined from the first heating. The glass transition temperature (*T*_g_) was taken at the half of the heat capacity jump and the melting temperature (*T*_m_) at the maximum of the endothermic peak. The degree of crystallinity was calculated according to the following relation:

$$x_{c}=\frac{\left(\Delta H_{m}-\Delta H_{\mathrm{cc}}\right)}{\Delta H_{m}^{0}},$$

where Δ*H*_m_ is the PLA melting enthalpy determined from the endothermic peak area, Δ*H*_cc_ is the cold crystallization enthalpy determined from the exothermic peak, and $\Delta H_{m}^{0}$ is the standard melting enthalpy of PLA taken equal to 93 J/g [13]. See the DSC curve of the sample from run 15 in Figure S4 in the SI. 

### 2.7. Optical Microscopy

The optical cross section microscopy pictures were obtained using an optical Keyence VHX1000 digital microscope (Bois-Colombes, France). The composites were cross-cut with a Kity 473 bandsaw and then the cross-cut surfaces were polished with a BTS800 belt and disc sander from Scheppach (Ichenhausen, Germany) before the analysis.

## 3. Results and Discussion

The RTM apparatus CIJECT III used in this study is composed of a tank where the monomer melts and is mixed with the catalyst, and a mold containing the glass fabrics where the polymerization of the matrix takes place to form the composite (Figure 1). As previously mentioned, in order to allow the injection in the mold, the mixture composed of the monomer and the catalyst must keep a low viscosity in the tank. Therefore, the polymerization must not be fully completed in this part of the apparatus, so the temperature should be kept as low as possible. The melting temperature of the l-lactide monomer (l-LA) being around 110 °C, the temperature of the tank was therefore set up at 120 °C. On the other hand, once the mixture is fully injected in the mold and the impregnation of the glass fabrics is complete, the polymerization must be as fast and efficient as possible in order to prevent the degradation of the PLLA matrix in the mold and in order to be compatible with industrial rates. The catalyst is also expected to give rise to polylactides with high molar mass to manufacture composites displaying high mechanical properties suitable for long-lifetime applications. Regarding the experimental constrains, we focused our studies on tin octoate, Sn(Oct)_2_, as the catalyst, as it is known to display one of the highest activities for the Ring Opening Polymerization (ROP) of cyclic esters, and in particular l-lactide, in bulk [14,15] or by reactive extrusion polymerization (a continuous process in a molten medium) [16]. At first, the activity of the catalyst was studied at the laboratory scale, in the conditions required by the RTM process, that is, in bulk at 120 °C to simulate the polymerization in the tank, and at 185 °C, the temperature where the catalyst presents suitable activity according to previous studies conducted by reactive extrusion process [17], to simulate the polymerization in the mold. The results are reported in Table 1.

Regarding the tests conducted at 120 °C, the tin octoate catalyst presents a relatively low activity at this temperature. Regardless of the monomer-to-catalyst ratio used, a maximum conversion of only 38% was observed after 40 min of reaction (run 1), which is suitable for the injection step during the RTM process. Concerning the lab-scale tests to simulate the polymerization in the mold after the injection step, which were conducted at 185 °C, the monomer loadings of 1000 and 2000 led to the best conversions (up to 87%) within short times (runs 6 and 7), allowing the production of materials with high molar masses (up to 149,000 g·mol^−1^). 

Regarding the results obtained for the preliminary small-scale polymerization tests, the monomer loadings of 1000 and 2000 equivalents seemed to be the most suitable for the RTM process, and were therefore used for the transposition tests.

The scale-up tests to the RTM were then conducted with two different types of molds: one mold with one imprint heated by conduction under press (capacity of 100 g of l-LA, mold 1) and one self-heating mold with two imprints (capacity of 400 g of l-LA, mold 2). The results are reported in Table 2. In order to ensure the feasibility of l-lactide polymerization in the RTM process with this catalyst, the reaction was initially carried out in the absence of reinforcement in the mold (runs 9–12). In all experiments, high conversions between 91% and 97% were at the laboratory scale (see Table S1 in the SI). One can observe that the presence of air reduced the activity of the catalyst, as a conversion of 90% was reached in 40 min in air instead of 12 min in argon. There was also a decrease of the molar masses, which were divided by more than half. In addition, it is noted that the results obtained under air were similar to those obtained during the RTM process. This may suggest that the atmosphere of the tank and/or the mold is not completely inert. 

Following these preliminary tests, we conducted reactions in the presence of glass fabrics (GFs), which were assumed to be inert towards the polymerization reaction (runs 13–15). Generally, one can observe that the presence of GF had little influence on the catalyst activity, regardless of the type of mold used (run 10 vs. 13 and 12 vs. 14), and that the monomer was fully obtained for polymerization times from 2 to 3 h, regardless of the monomer-to-catalyst ratio (1000 or 2000) and the type of mold. The reaction times were much longer than those observed for the preliminary polymerization tests, due to the absence of stirring in the RTM mold in opposition to the small-scale experiments conducted in flasks, inducing a lack of diffusion of the monomer to the catalyst. In addition, the molar masses of the obtained polylactides in the RTM process were lower than the expected ones for such high conversions and also in regard to the preliminary tests (18,300–36,600 g·mol^−1^ for runs 9–12 vs. 121,100–149,200 g·mol^−1^ for runs 6 and 7). This could be due to the fact that, even if the polymerization reactions were conducted under nitrogen in the RTM process, the atmosphere in the tank and the mold was not as pure as that in the flask for the reactions conducted at the lab-scale and prepared in a glove box. To verify this hypothesis, polymerization tests were performed in air converted in all cases. In the particular case of run 15, the test was conducted under dynamic vacuum in the mold, in order to maintain the mold tightly closed and ensure an atmosphere as pure as possible to enhance the polymerization rate. This type of resin transfer molding in which dynamic vacuum is applied at the exit vent would qualify as Vacuum-Assisted Resin Transfer Molding (VARTM) process [18]. In this case, the molar mass of the PLLA matrix increased to 78,600 g·mol^−1^ (run 15), which is closer to the results obtained for the polymerization carried out at the lab scale under inert atmosphere in a glove box. Regarding the thermal characteristics of the PLLA matrices obtained in the presence or absence of glass fabrics, the samples synthesized in mold 2 were semi-crystalline (crystallinity from 45% to 58%; runs 11, 12, 14, and 15), whereas those obtained using mold 1 were quasi-amorphous (runs 9, 10, and 13). This can be explained by the different types of cooling applied at the end of the polymerization reaction, which is linked to the type of mold employed. Mold 1, which was heated by conduction under press, was cooled using a chiller within 20 min, whereas with mold 2 the cooling was ensured by the surrounding atmosphere of the room, and thus was much slower. Regarding the melting of the crystalline phase, a shoulder in the melting peak was observed in some cases (runs 9–13, Figures S4 to S8 in the SI). This observation has been reported previously and is assigned to a phase transformation phenomenon [19].

**Figure 2:**
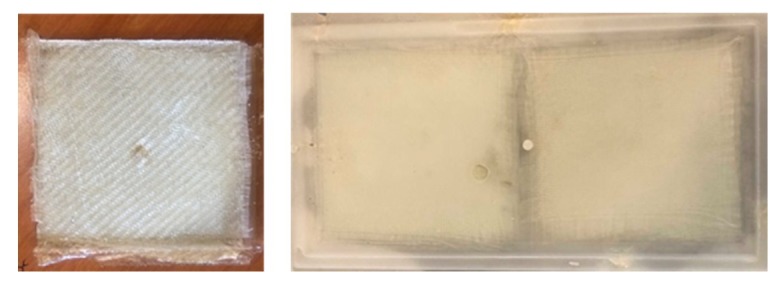
PLLA matrix composites obtained with molds 1 (run 13, left) and 2 (run 15, right), respectively.

**Figure 3:**
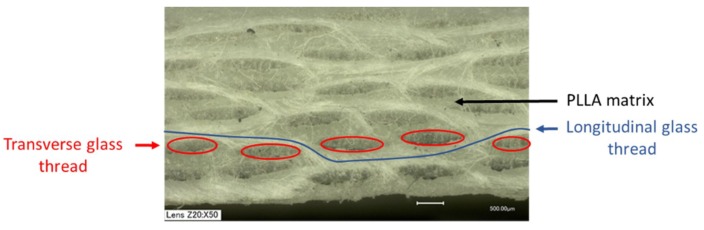
Optical microscopy picture of PLLA matrix composite (run 15).

Finally, regardless of the type of mold used, the filling of the mold cavity was efficient, as seen by observing the resulting glass fabric/PLLA composite prototypes obtained (Figure 2). Moreover, the analysis by optical microscopy of the surface of samples displaying the highest molar mass (run 15) after being cross-cut showed a good impregnation of the reinforcement by the matrix, as no lack of material nor bubbles were visible (Figure 3).

## 4. Conclusions

In this paper, we have reported the first example of the synthesis of glass fabric/PLLA composites by T-RTM, via in situ polymerization of the matrix with tin octoate as the catalyst. The atmosphere in the RTM apparatus played a decisive role in obtaining a polylactide composite matrix of high molecular mass. Indeed, the best result—namely, a composite with a matrix molecular mass of 78,000 g·mol^−1^—was obtained when the process was carried out under dynamic vacuum, which suggested that a VARTM process can be more appropriate for this type of synthesis. In the continuation of this study, further RTM tests with other metal-based catalysts and reinforcements of various nature will be carried out, in addition to stress tests accounting for the mechanical properties of the PLLA-based composites obtained.

## Figures and Tables

**Table 1:** Bulk polymerization of l-lactide (l-LA) with Sn(Oct)_2_ at the laboratory scale *^a^*.

Run	[L-LA]/[Sn]	T (°C)	Time (min)	Conversion *^b^* (%)	*M*_n_*^c^* (g·mol^−1^)	*Ð ^c^* (g·mol^−1^)
1	500	120	40	38	68,700	1.08
2	1000	120	40	34	85,300	1.11
3	2000	120	60	31	124,300	1.24
4	5000	120	100	11	90,400	1.08
5	500	185	3	65	68,000	1.43
6	1000	185	6	83	121,100	1.74
7	2000	185	12	87	149,200	1.31
8	5000	185	10	31	131,140	1.34

*^a^* Experimental conditions: under argon in a glove box, mass of l-LA = 1 g. *^b^* determined by ^1^H NMR in CDCl_3_. *^c^* determined by SEC in THF at 40 °C, refractive index (RI) detection, *M*_n_ corrected by the coefficient 0.58 for PLLA.

**Table 2:** Preparation of poly(l-lactide) (PLLA) matrices and PLLA composites with glass fabrics by RTM process *^a^.*

Run	Mold *^b^*	[L-LA] /[Sn]	Glass Fabrics (%)	Time (h)	Conversion *^c^* (%)	*M*_n_*^d^* (g·mol^−1^)	*Ð ^d^* (g·mol^−1^)	*T*_g_*^e^* (°C)	*T*_m_*^e^* (°C)	X_c_ *^e^* (%)
9	1	1000	-	2	97	33,200	1.15	49	169	2
10	1	2000	-	3	96	36,600	1.29	51	169	4
11	2	1000	-	2	96	22,300	1.28	no*^g^*	176	45
12	2	2000	-	3	91	18,300	1.25	no	166	58
13	1	2000	35	3	98	30,600	1.24	52	169	5
14	2	2000	20	2.5	99	35,300	1.25	no	169	55
15 *^g^*	2	2000	20	3	99	78,600	1.33	no	173	47

*^a^* Experimental conditions: Sn(Oct)_2_ as the catalyst, under nitrogen, *T* = 185 °C, mass l-LA = 100 g for mold 1 and 400 g for mold 2. *^b^* mold 1: one imprint 120 mm × 120 mm × 5 mm; mold 2: two imprints 120 mm ×120 mm × 5 mm. *^c^* determined by ^1^H NMR in CDCl_3_. *^d^* determined by SEC in CHCl_3_ at 20 °C, MALLS method with dn/dc = 0.023. *^e^* determined by DSC from the 1st heating, calculation of crystallinity degree: *X*_c_ = [(Δ*H*_m_ − Δ*H*_c_)/ (Δ*H*^0^_m_ = 93)]. *^f^* no = not observed. *^g^* experiment conducted under dynamic vacuum (vacuum-assisted RTM, VARTM).

## Supplementary Materials

The supplementary materials are available online at http://www.mdpi.com/2073-4360/11/2/339/s1.
