# Supplementary material for: Preparation of Glass Fabric/Poly(l-lactide) Composites by Thermoplastic Resin Transfer Molding
Source: Polymers (Basel). 2019 Feb 15;11(2):339. doi: 10.3390/polym11020339 (PMC6419152; doi:10.3390/polym11020339)
Supplement: Supplementary file 1 [file polymers-11-00339-s001.pdf]

Supporting Information for

Preparation of glass fabric/poly(L-lactide) composites by Thermoplastic Resin Transfer Molding

Elodie Louisy [1,2], Fabienne Samyn [1], Serge Boubigot [1], Gaëlle Fontaine [1] and Fanny Bonnet [1*]

[1] Univ. Lille, CNRS, INRA, ENSCL, UMR 8207 - UMET - Unité Matériaux et Transformations, F-59000 Lille, France
[2] Univ. Lille, CNRS, INRA, ENSCL, UMR 8181 - UCCS - Unité Catalyse et Chimie du Solide, F-59000 Lille, France

Email : fanny.bonnet@ensc-lille.fr.

Content

Figure S1. RTM tank, mold 1 and mold 2 of the RTM apparatus

Table S1. Comparative synthesis of PLLA at the laboratory scale under air or argon

Figure S2. ^{1}H NMR spectrum ($CDCl_3$) of poly(L-lactide) matrix for conversion determination

Figure S3. SEC trace of polylactide matrix in run 15

Figure S4 to S10. DSC curve of polylactides

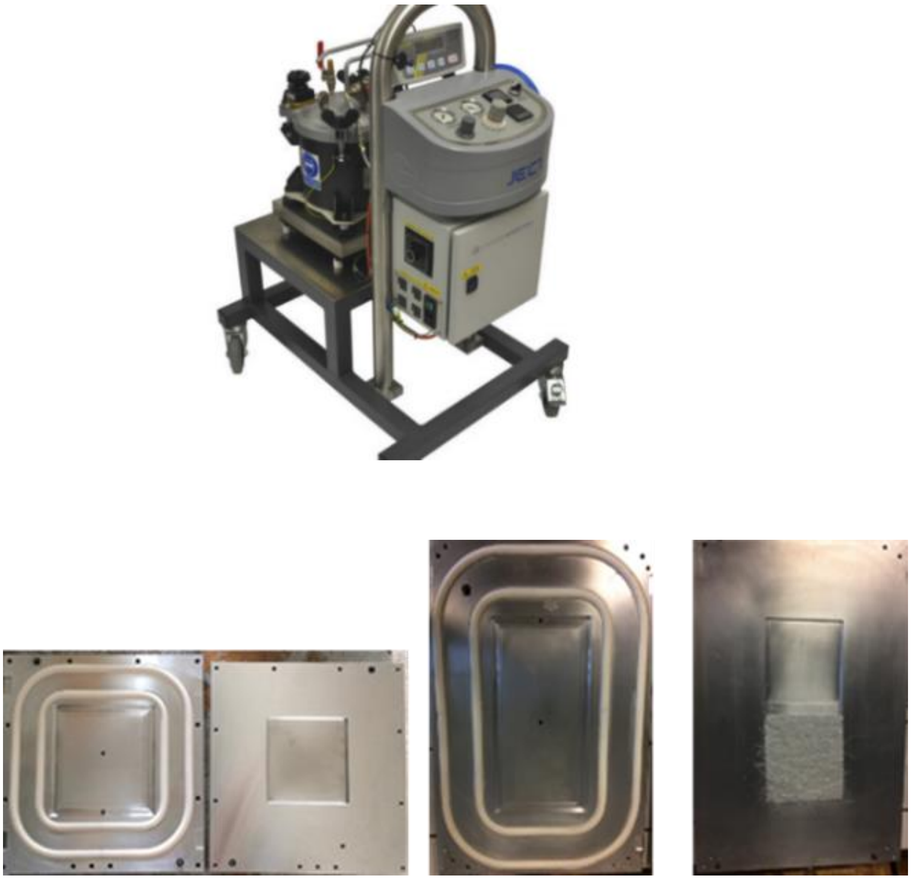

Figure S1. RTM tank, mold 1 and mold 2 of the RTM apparatus

Table S1. Comparative synthesis of PLLA at the laboratory scale with $Sn(Oct)_2$ under air or argon [a]

Run	Atmosphere	Time (min)	Conversion [b] (%)	M_n [c] ($g.mol^{-1}$)	Đ [c] ($g.mol^{-1}$)
1	Ar	12	87	149 200	1.30
2	Air	40	90	57 300	1.57

[a] Experimental conditions: mass of L-LA = 1g, [L-LA]/[Sn] = 2000, 185°C, [b] determined by ^1H NMR in $CDCl_3$. [c] determined by SEC in THF at 40°C, RI detection, M_n corrected by the coefficient 0.58 for PLLA.

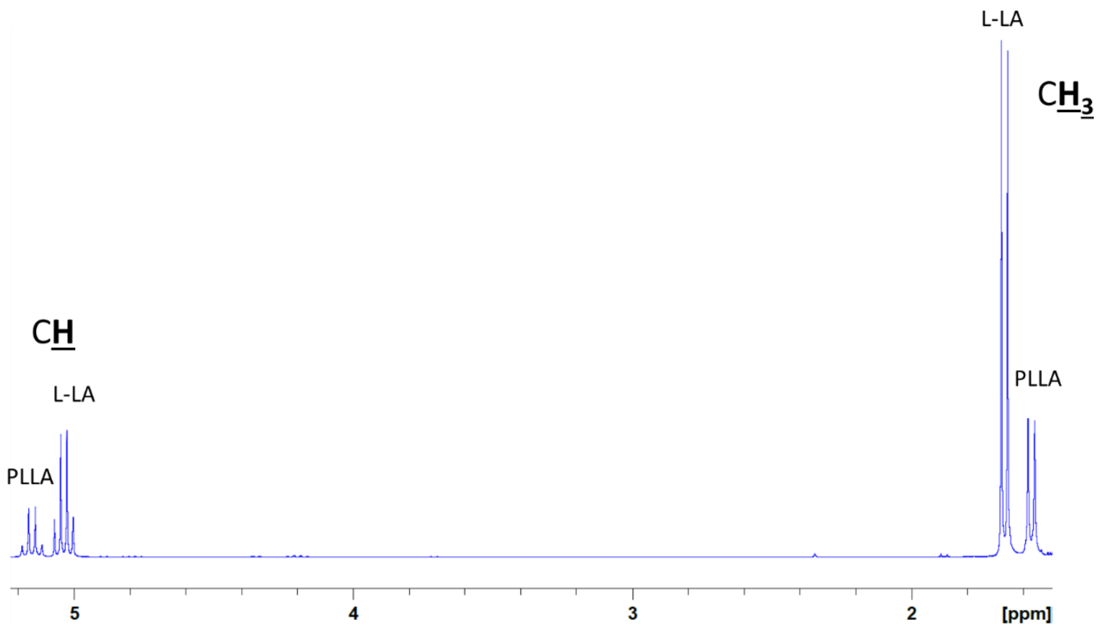

Figure S2. ^{1}H NMR spectrum ($CDCl_3$) of poly(L-lactide) matrix in run 2 table S1. Conversion of the polymerization reaction was determined by integration of the C*H* signals relative to L-LA and PLLA at 5.03 and 5.15 ppm respectively.

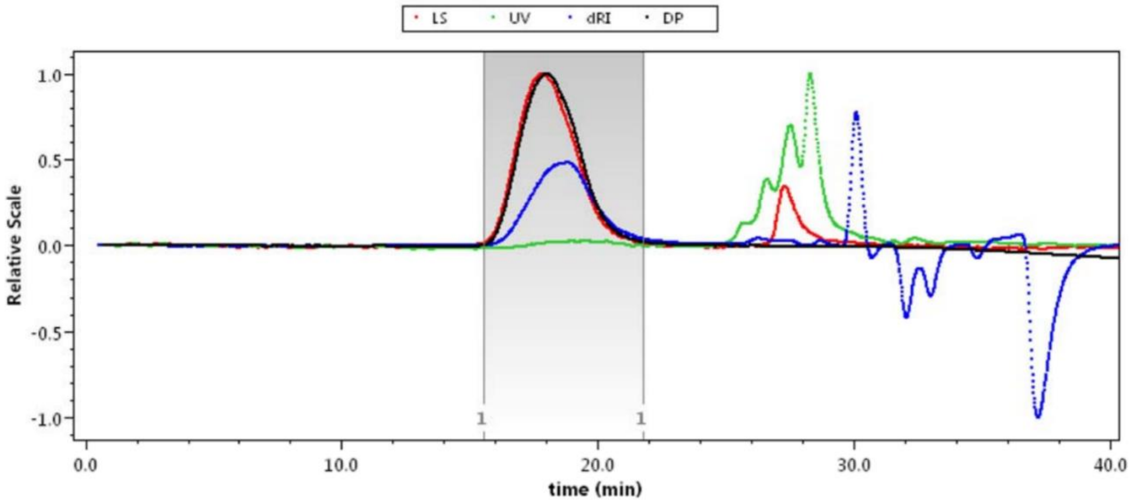

Figure S3. SEC trace of polylactide matrix in run 15. Detectors: Light scattering (LS), Ultraviolet (UV), Refractive index (RI), Differential pressure (DP).

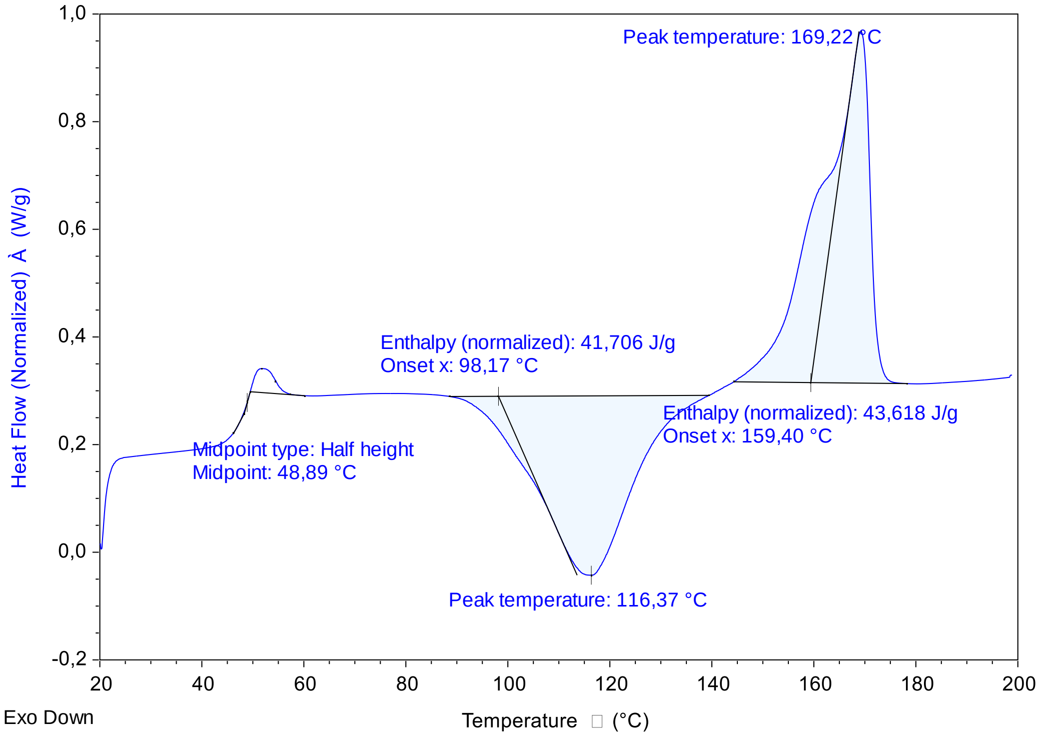

Figure S4. DSC curve of polylactide matrix in run 9 (1st heating)

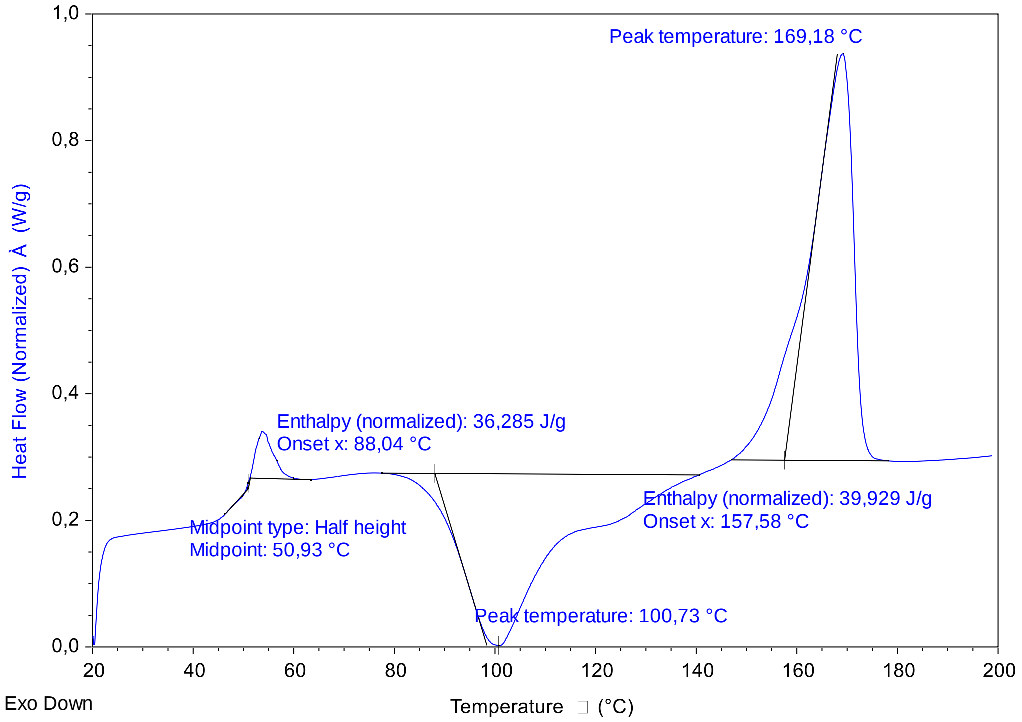

Figure S5. DSC curve of polylactide matrix in run 10 (1st heating)

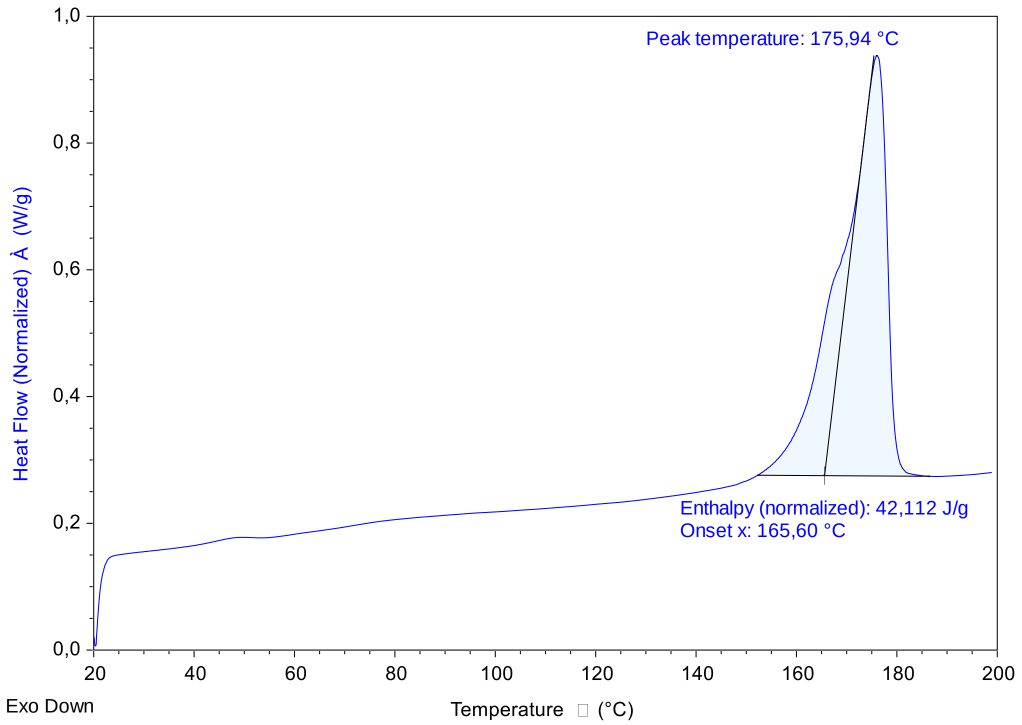

Figure S6. DSC curve of polylactide matrix in run 11 (1st heating)

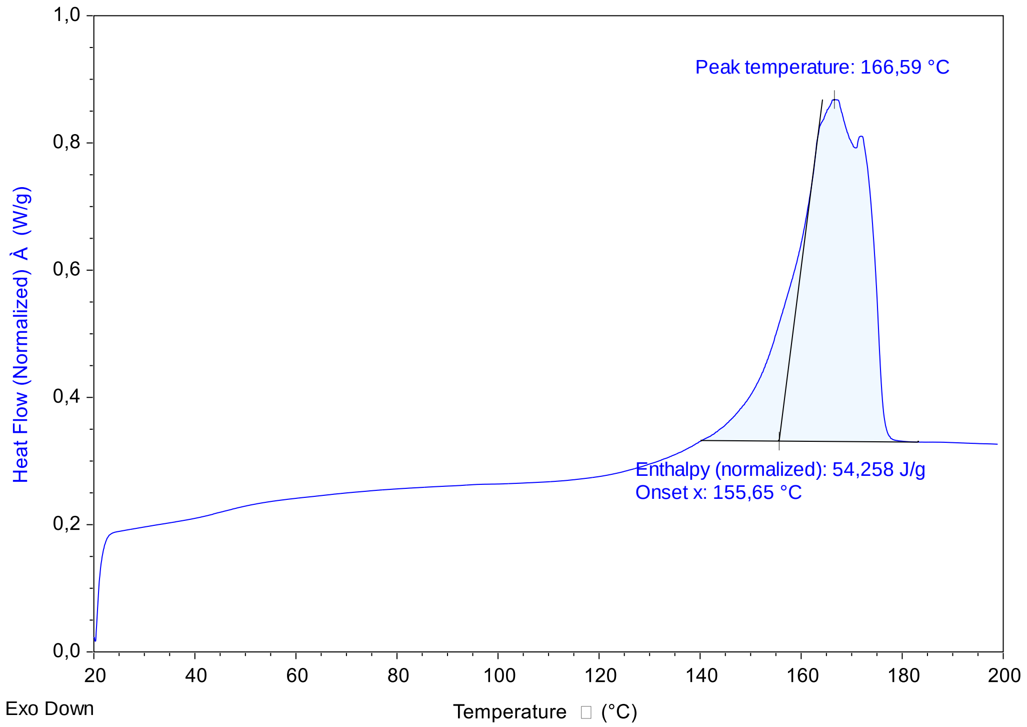

Figure S7. DSC curve of polylactide matrix in run 12 (1st heating)

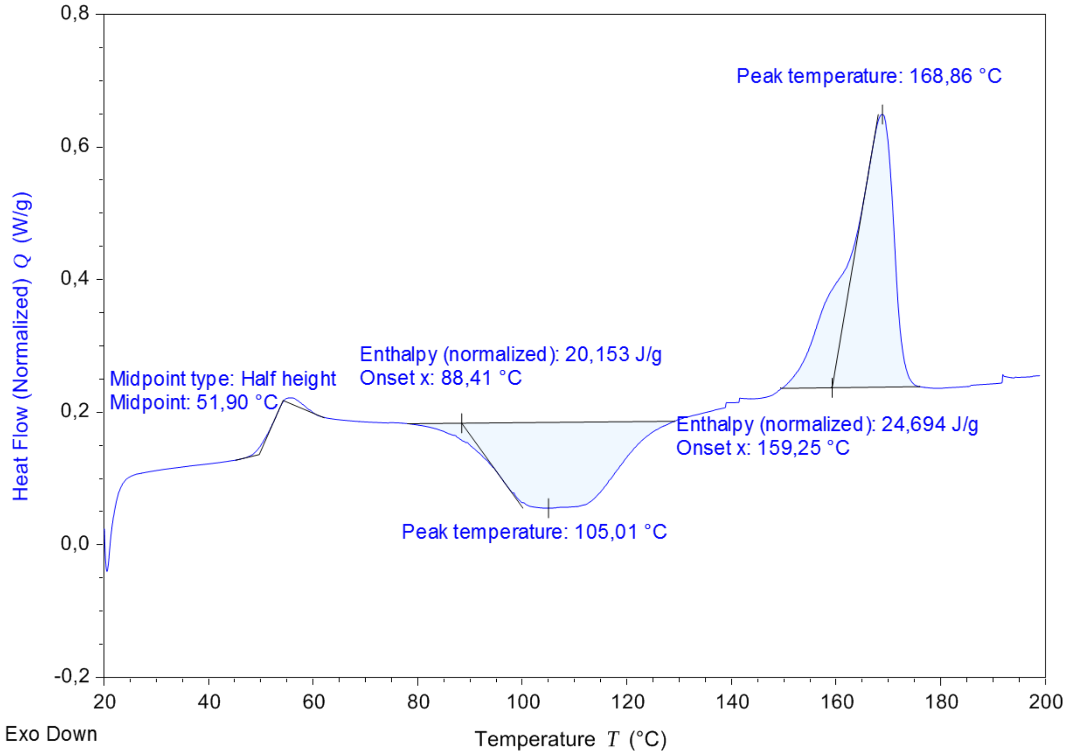

Figure S8. DSC curve of polylactide matrix in run 13 (1st heating)

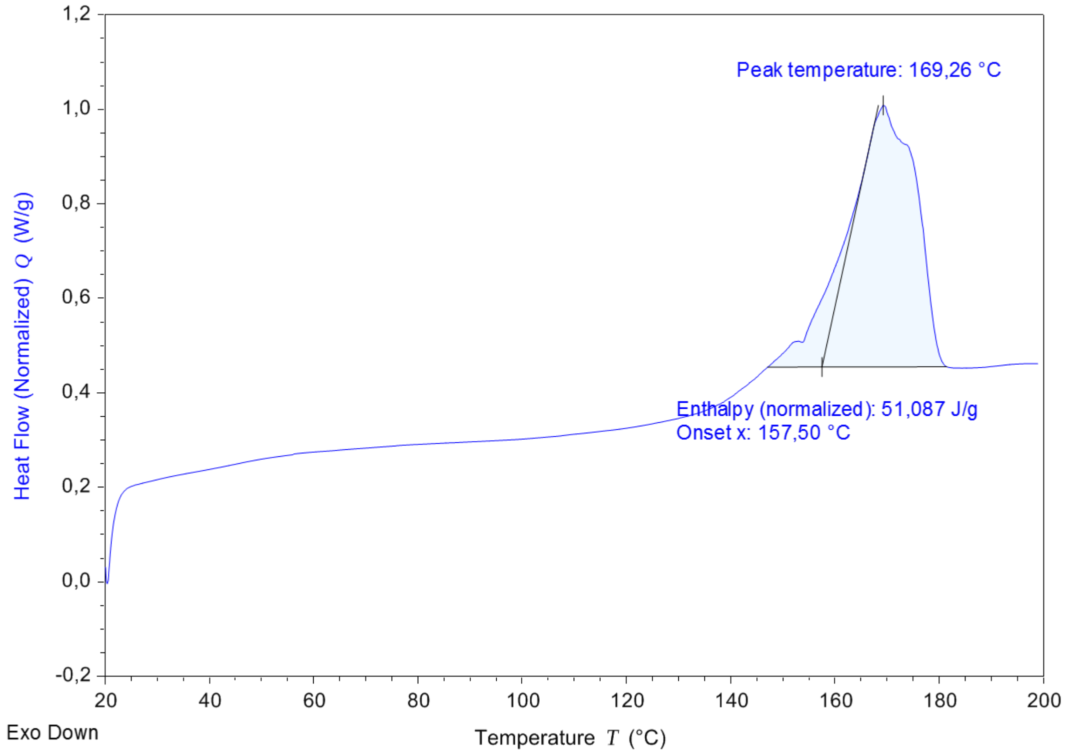

Figure S9. DSC curve of polylactide matrix in run 14 (1st heating)

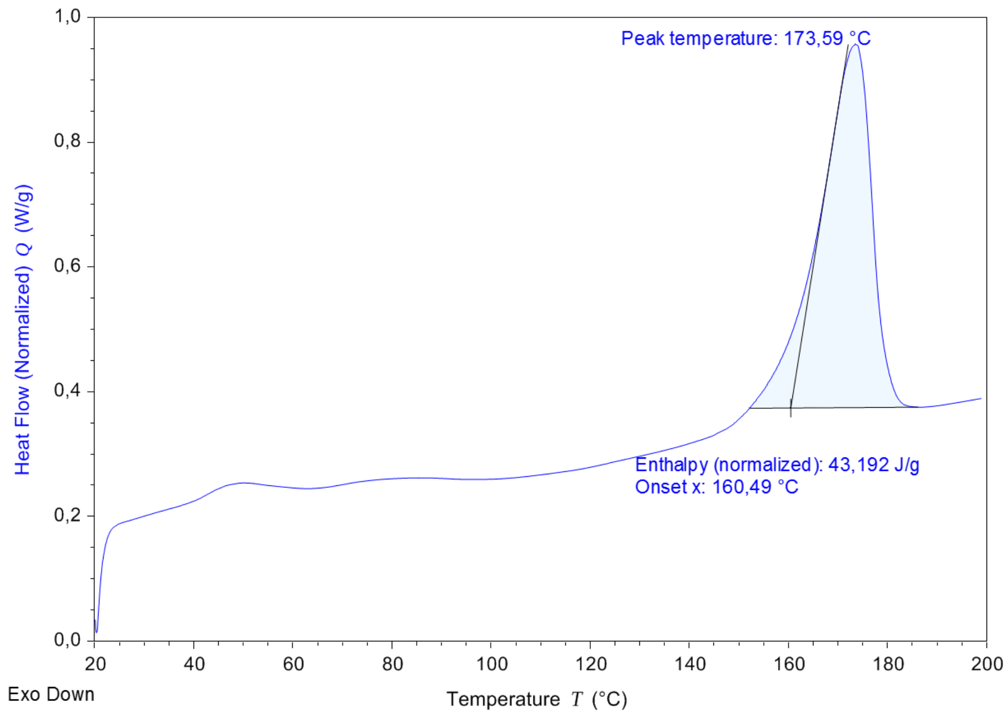

Figure S10. DSC curve of polylactide matrix in run 15 (1st heating)
